# Evaluation of Antibody Response to Various Developmental Stage Specific Somatic Antigens of *Paramphistomum epiclitum* in Goats

**DOI:** 10.1155/2014/505484

**Published:** 2014-06-05

**Authors:** Jyoti, A. Prasad, Nirbhay Kumar Singh

**Affiliations:** ^1^Department of Veterinary Parasitology, Guru Angad Dev Veterinary and Animal Sciences University (GADVASU), Ludhiana 141004, India; ^2^Division of Parasitology, Indian Veterinary Research Institute, Izatnagar 243122, India

## Abstract

Electrophoretic analysis of various developmental stage specific somatic antigens of *Paramphistomum epiclitum* (Digenea: Paramphistomidae), namely, metacercariae (McAg), immature intestinal flukes (ImIAg), immature ruminal flukes (ImRAg), and adult flukes (AAg), was done by native polyacrylamide gel electrophoresis. Result revealed presence of 3 (range 15.2–40.3 kDa), 13 (9.3–121.2 kDa), 14 (9.3–169.3 kDa), and 15 (8.0–169.3 kDa) polypeptides in McAg, ImIAg, ImRAg, and AAg, respectively. With an aim to identify a suitable immunodiagnostic antigen for early diagnosis of amphistomosis, the IgG antibody response to various developmental stage antigens in goats experimentally infected with metacercariae of *P. epiclitum* was evaluated by ELISA. The highest OD values were recorded with ImIAg which ranged between 0.23 and 0.55 with a significant increase from the 2nd week till 8th week of infection with a peak at 6th week. The analysis of statistical significance using a one-way analysis of variance with multiple pair wise comparisons revealed that IgG response was significantly higher with all antigens (*P* < 0.01) except McAg (*P* > 0.05) with a maximum mean difference of 0.1838 in comparison to control with ImIAg, thus, indicating that ImIAg which could be further exploited for its potential is a candidate for immunodiagnostic antigen for early diagnosis of amphistomosis.

## 1. Introduction 

*Paramphistomum epiclitum *(Digenea: Paramphistomidae) is a gastric trematode affecting ruminants with a wide range of geographic distribution and is prevalent in several states of India [1–4]. The life cycle is indirect and sexually mature fluke in the rumen releases the eggs along with faeces which hatch in water into ciliated miracidia. The miracidia then enters the body of an intermediate host (snail), in which it develops to produce cercariae and encyst to become metacercariae on aquatic plants or other suitable substrata. Upon ingestion of viable metacercariae once inside the duodenum and jejunum, their cysts are removed, they penetrate the intestinal wall by actively destroying the mucosa and then migrate to the rumen, where they grow into adult [5]. The adult stages generally have low pathogenicity while the migrating immature ones cause severe pathology and even kill the host in heavy infections as they are attached to the wall of the small intestine causing hemorrhagic inflammation, characterized by focal infiltration of macrophages and lymphocytes in the lamina propria [6]. The injury caused in ruminants severely affects production, since these parasites cause a lower feed conversion, a loss of weight, and/or a decrease in milk production, responsible for severe economic losses [7].

The diagnosis of most of the helminth diseases is done through the demonstration of its eggs in the faeces of the infected animal. However, in case of amphistomosis as the infected animals exhibit clinical symptoms much before passing of eggs in the faeces (disease syndrome is caused by immature stages and has a relatively long prepatent period), coproscopic analyses often results in misdiagnosis and could not be used in early diagnosis [8]. Thus, immunological diagnosis can prove to be an important tool for early diagnosis of amphistomosis which is essential for prompt treatment before irreparable damage to the rumen and small intestine occurs [9]. Research on several parasitic trematode infections has shown that the immune system is stimulated to produce specific antibodies against the parasites and its detection by means of indirect enzyme-linked immunoassays can prove to be an effective tool for early disease diagnosis [10–12].

Regarding immunodiagnosis of amphistomosis limited work has been done utilizing either adult worm somatic antigen [13–17], excretory/secretory antigen [11, 18] or coproantigen [12] as antigen. It is also well known that helminth parasites during the course of development undergo antigenic polymorphism which induces drastic alterations in immune response, so use of these different developmental stage antigens is very important in immunodiagnosis. Further, as life cycle of* P. epiclitum* involves various developmental stages, therefore, the antigens derived from these stages may exhibit different immune response in the host. Hence, study of immune response against the various developmental stage antigens would be helpful in identification of sensitive immunodiagnostic antigen for early diagnosis of prepatent amphistomosis. In the present study, antigens derived from different developmental stages of the parasite, namely, metacercariae, immature intestinal, immature ruminal, and adult ruminal flukes, have been used to evaluate the IgG response in goats experimentally infected with* P. epiclitum*.

## 2. Materials and Methods

### 2.1. Collection of Metacercariae

**Figure 1:**
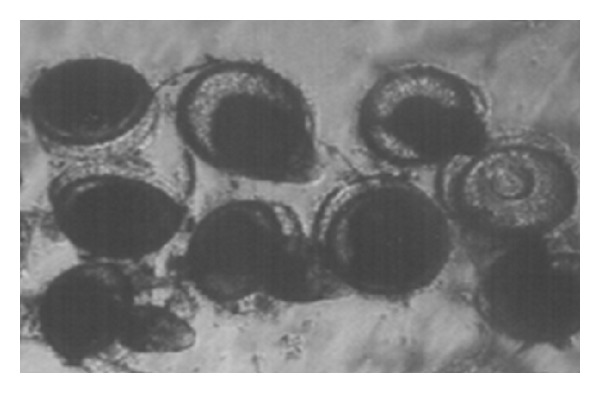
*In vitro* excystment of metacercariae of* P. epiclitum.*

**Figure 2:**
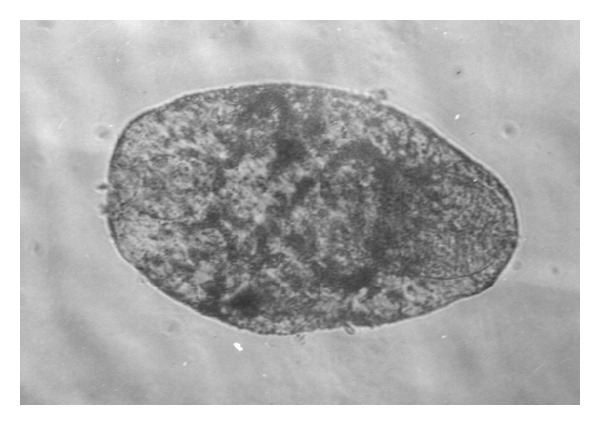
Juvenile fluke of* P*.* epiclitum*.

*Indoplanorbis exustus* snails were collected from ponds of villages nearby Indian Veterinary Research Institute, Izatnagar, India, during the monsoon and post-monsoon seasons, maintained in the laboratory in glass troughs and fed fresh spinach leaves. Snails were screened individually for* P. epiclitum* infection by exposure to artificial light (40-watt candescent bulb) which caused emergence of cercariae within an hour. Infected snails were sorted out and cercariae emerging out from them encysted as metacercariae on yellow polythene sheets [19]. The metacercariae were stored in triple distilled water at room temperature (25–28°C) till further use for antigen preparation and setting up of experimental infection in goats. Before use, the viability of metacercariae was determined on the basis of motility of juveniles within the cyst as observed under stereoscopic microscope and* in vitro* excystment of viable metacercariae as per the method described by Jyoti et al. [20] (Figure 1). Briefly, 100 metacercariae were taken in a small petridish and 5 mL N/20 HCl and equal volume of solution containing 0.8% NaCl and 1% NaOH was added and incubated at 44°C for 10 min. Then, L-cysteine HCl @ 4 mg/mL was added and kept at room temperature for 30 min. Later, 10 mL of 2% solution of bile salts was added and incubated at 44°C for 10–12 h. The freshly excysted juvenile flukes were maintained in Ringer's Locke solution at room temperature (Figure 2).

### 2.2. Collection of Parasite

**Figure 3:**
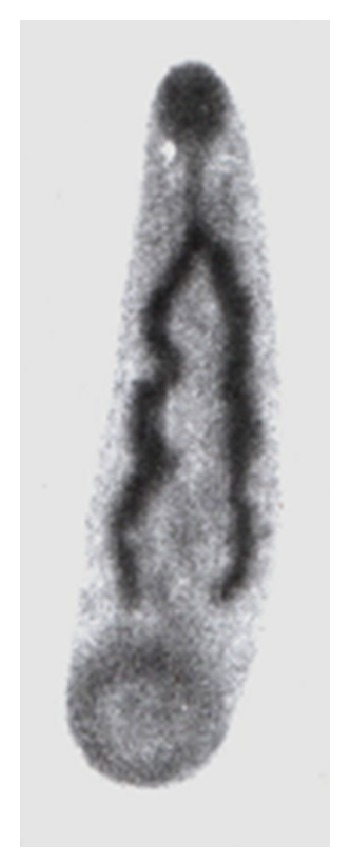
Immature* P*.* epiclitum*.

**Figure 4:**
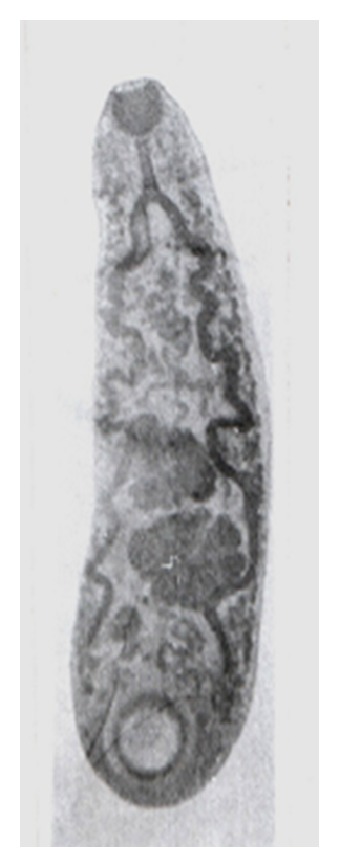
Mature* P*.* epiclitum*.

Adult and immature stages of* P. epiclitum* were collected from rumen and small intestine from the gastrointestinal tracts of goats obtained from local abattoir. The parasites were collected in 0.01 M phosphate buffered saline, pH 7.2 (PBS), thoroughly washed with PBS and stored at −20°C for antigen preparation. The identification of the parasite was confirmed after preparing permanent slides using standard keys [21] (Figures 3 and 4). Briefly, the flukes were placed between two glass slides, flattened, and tied with a piece of thread and then placed in 70% alcohol for 24 hours. The flukes were dislodged from the slides and subjected to overnight Borax Carmine staining, followed by destaining in 2% acid alcohol, graded dehydration, clearing in clove oil, and mounting in DPX (distyrene plasticizer and xylene).

### 2.3. Preparation of Lifecycle Stage Specific Somatic Antigens of* P. epiclitum*

Somatic antigens derived from various developmental stages, namely, metacercariae (McAg), immature intestinal flukes (ImIAg), immature ruminal flukes (ImRAg), and adult flukes (AAg), were prepared as previously described by Bennett et al. [22] and Guobadia and Fagbemi [23] with slight modifications.

Briefly, for preparation of McAg approximately 10,000 metacercariae were washed with 0.01 M PBS, homogenized, sonicated for six cycles at 16 *μ* peak for 10 sec with 30 sec interval at 4°C, and centrifuged at 11,750 ×g for 30 min at 4°C and supernatant was collected. The supernatant was filtered using 0.22 *μ*m syringe filter (Millipore, Billerica, MA, USA); a cocktail of protease inhibitors (Sigma-Aldrich, St. Louis, IL, USA) was added and stored at −20°C.

Similarly, immature* P. epiclitum* flukes obtained from the small intestine and rumen of goats were utilized for preparation as ImIAg and ImRAg, respectively. Briefly, the immature flukes were homogenized in 0.01 M PBS in a Teflon coated homogenizer at 4°C, sonicated for five cycles at 8 *μ* peak for 2 min with 1 min interval at 4°C, and centrifuged at 11,750 ×g for 1 hr at 4°C. Supernatant was collected and pooled, filtered using 0.22 *μ*m syringe filter (Millipore, Billerica, MA, USA); a cocktail of protease inhibitors (Sigma-Aldrich, St. Louis, IL, USA) was added and stored at −20°C. Further, adult flukes collected from the rumen of goats were utilized for the preparation of AAg as per the method described above.

### 2.4. Protein Estimation

Protein concentrations of all the antigens were estimated as per the method of Lowry et al. [24], using bovine albumin fraction V as standard.

### 2.5. Polyacrylamide Gel Electrophoresis (PAGE)

Native PAGE of various lifecycle stage specific somatic antigens was performed in 5–15% gradient resolving and 5% stacking polyacrylamide gels using a discontinuous system as described by Laemmli [25]. For determination of molecular weight a prestained protein marker (14.3–97.4 kDa) (Bangalore Genei, Bengaluru, India) was also subjected to electrophoresis in a vertical electrophoresis system (Bangalore Genei, Bengaluru, India) at 120 V constant voltage. The gels were subjected to Coomassie brilliant blue (CBB) stain for 4 h, followed by destaining and scanned by gel documentation system (Syngene, Frederick, MD, USA).

### 2.6. Experimental Infection of Goats

Six healthy male goats of about six months of age were procured from the Sheep and Goat Farm, LPM Division, IVRI, Izatnagar. Faecal sample of all animals were examined using sedimentation and floatation techniques to confirm their* P. epiclitum* naive status. Four goats were given a dose of 3,500 viable metacercariae of* P. epiclitum* orally after 12 h of fasting and two were maintained as control. The animal experimentations were conducted in compliance with the ethical considerations and guidelines issued by CPCSEA/Institutional Animal Ethics Committee (IAEC) on laboratory animals.

### 2.7. Collection of Sera Samples

Blood samples were collected from the jugular vein of all animals from day zero to eight weeks postinfection at weekly interval. The sera were separated, aliquoted in 1.5 mL, and stored at −20°C after adding thiomersal (10 mg/mL) @ 5 *μ*L/mL.

### 2.8. Enzyme Linked Immunosorbant Assay (ELISA)

For the analysis of the humoral IgG response, ELISA was carried out with all four somatic antigens following the method of Njau and Nyindo [26] with slight modifications. Flat-bottomed polystyrene Greiner 96-well plates (Sigma-Aldrich, St. Louis, IL, USA) were coated with 100 *μ*L of antigen per well in carbonate bicarbonate coating buffer (pH 9.6) (protein concentration 10 *μ*g per mL) and incubated at 4°C overnight. Then the plates were washed thrice with PBS containing 0.1% Tween-20 (washing buffer), blocked with 150 *μ*L of 5% skimmed milk in PBS, and incubated at room temperature for 1 h. The washing step was repeated again before adding 100 *μ*L of 1 : 250 dilutions of the primary sera collected from experimentally infected and control animals in PBS (in quadruplet wells) and kept at 37°C for 2 h. Following washing, again 100 *μ*L of 1 : 5000 diluted rabbit anti-goat IgG conjugated with horseradish peroxidase (Bangalore Genei, Bengaluru, India) in PBS was added and incubated for 2 h at 37°C. After washing, the reaction was developed by adding 100 *μ*L of substrate solution containing 40 mg O-phenyldiamine (Sigma-Aldrich, St. Louis, IL, USA) in 100 mL phosphate citrate buffer (pH 5.0) and 40 *μ*L hydrogen peroxide and incubated in dark at room temperature for 30 min. The colour reaction was stopped by the addition of 3 N sulphuric acid and optical density values were read at 492 nm using Titertek multiskan plate reader (Labsystems, Finland). The control wells for primary antibody, secondary antibody, substrate, and antigen were maintained in the plate. The analysis of statistical significance using a one-way analysis of variance (ANOVA) with group multiple comparisons was done using the Tukey's test (GraphPad Prism 4 software).

## 3. Results

### 3.1. *In Vitro* Excystment of Metacercariae

The percent of excystment in metarcercarie stored for 2, 10, 20, and 60 days was 95, 87, 85, and 40%, respectively (Figure 1). The freshly excysted juvenile flukes were maintained in Ringer's Locke solution at room temperature for 5-6 days (Figure 2).

### 3.2. Protein Concentration of Antigens

Protein concentrations of various stage specific somatic antigens, that is, McAg, ImIAg, ImRAg, and AAg, were 1.2, 4.6, 16.0, and 13.5 mg/mL, respectively.

### 3.3. Native PAGE

**Figure 5:**
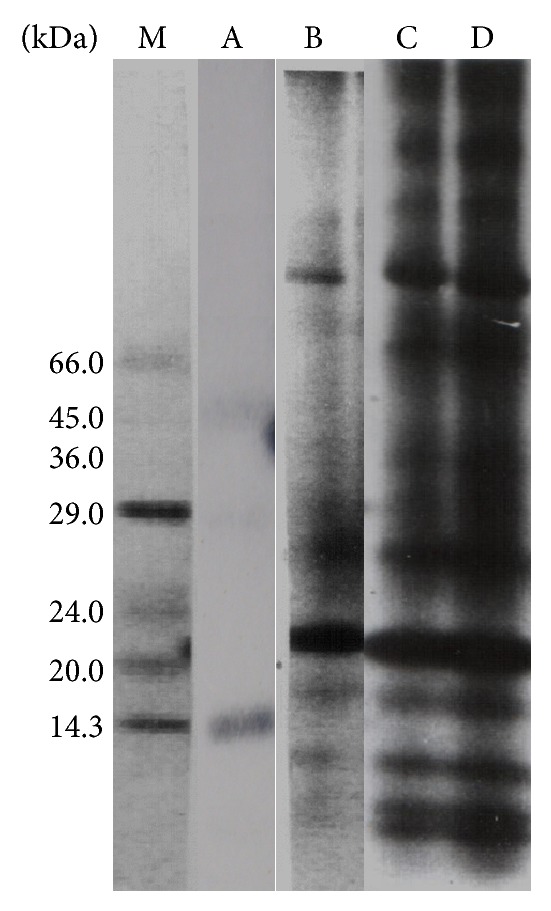
Native-PAGE profile of various stage specific antigens of* P. epiclitum.* Lane M: molecular weight marker; Lane A: metacercarial antigen (McAg); Lane B: immature intestinal fluke antigen (ImIAg); Lane C: immature ruminal fluke antigen (ImRAg); Lane D: adult fluke antigen (AAg).

Electrophoretic analysis of various somatic antigens revealed 3 polypeptides of 40.3, 31.5, and 15.2 kDa in McAg. Further, ImIAg, ImRAg, and AAg showed 13, 14, and 15 polypeptides in range of 9.3–121.2, 9.3–169.3, and 8.0–169.3 kDa, respectively, by native PAGE. The 169.3 kDa polypeptide was found lacking in ImIAg but was present in the other two somatic antigens whereas polypeptide of 8.0 kDa was recorded only in AAg. Among all the antigens 13 polypeptides of mol. wt. 121.2, 103.2, 79.1, 65.0, 43.0, 36.9, 32.7, 26.2, 18.9, 15.4, 11.8, 10.0, and 9.3 kDa were found common (Figure 5).

### 3.4. ELISA with Various Stage Specific Antigens

**Figure 6:**
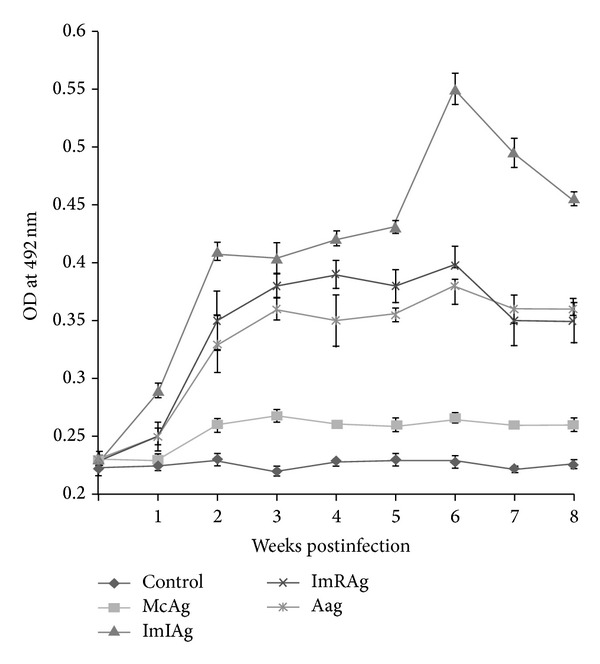
Antibody response by ELISA using various stage specific antigens of* P. epiclitum.*

The humoral IgG response generated in goats experimentally infected with metacercariae of* P. epiclitum* was monitored through ELISA by using all four stage specific somatic antigens (McAg, ImIAg, ImRAg, and AAg) up to 8 weeks post infection (wpi). A marked variation in antibody response was recorded with various antigens. A negligible antibody response was recorded when McAg was used for ELISA as the OD values in the experimentally infected goat sera were almost similar to noninfected control up to 8 wpi. The OD values recorded with ImIAg antigen in experimental sera samples ranged between 0.23 and 0.55 which was more than double as compared to control. There was a marked increase in the antibody response of infected group from the 2nd week and was maintained till 8th week of infection with a peak at 6th wpi. Similarly, when ImRAg was employed as antigen the OD values ranged between 0.23 and 0.40 with experimental sera and the trend of response was similar to ImIAg. For AAg the OD value ranged between 0.23 and 0.38 with highest values at 6 wpi (Figure 6). Hence, all the antigens except McAg detected a marked IgG response in the experimental sera as early as 2nd week post infection. In ANOVA with multiple pair wise comparisons the IgG response was significantly higher with all antigens (*P* < 0.01) except McAg (*P* > 0.05) with a maximum mean difference of 0.1838 in comparison to control with ImIAg (Table 1).

## 4. Discussion

Amphistomosis is a neglected ruminant disease causing high morbidity and mortality in tropical and subtropical countries resulting in great economic losses. Prevalence of various species of amphistome parasites among the livestock has been reported from a number of states of India [1–4]. The rumen amphistomes particularly* Gastrothylax crumenifer* and* Paramphistomum epiclitum* have marked seasonality in egg production [27] which makes the task of conventional diagnosis more difficult during routine parasitological investigation involving detection of eggs in faecal samples. Although pioneer work on the life cycle of various amphistomes has been worked out in this country, immunodiagnosis was neglected.

The electrophoretic profile of various stage specific somatic antigens of* P. epiclitum* was studied which showed a difference in the polypeptide profile of various antigens in terms of their differential electrophoretic mobility on native PAGE gel. Although the current study seems to be first of its kind reporting the polypeptide profile of various antigens of* P. epiclitum* of goats but in similar studies SDS-PAGE analysis of crude somatic adult antigen of* P. epiclitum* showed 12 polypeptides in the range of 19.9–85.1 kDa [28] and 14 polypeptides in the range of 14.1–95.5 kDa [15]. Further, the presence of 13 common polypeptides among the various stage specific antigens supports the view that some major polypeptides are conserved during evolution, whereas the variation in the polypeptide profile may be due to genomic heterogeneity [29].

The probability of diagnosis of animals infected with amphistomes is more by detecting the circulating antibodies in the sera samples of infected animals as compared to the functional/structural antigen of the parasite by ELISA [13]. It is interesting to note that studies on immunodiagnosis of amphistomosis have been mostly based on either adult worm somatic antigen [13–17], excretory/secretory antigen [11, 18], or coproantigen [12]. A number of investigators have tried to develop specific serodiagnostic tests for early detection of a related trematode species,* Fasciola*, in animals [10, 30–32], whereas in the current study the antigens were derived from different developmental stages of the parasite, namely, metacercariae, immature intestinal flukes, immature ruminal flukes, and adult ruminal flukes, to evaluate the IgG response in goats experimentally infected with* P. epiclitum*. The polypeptide profile changes recorded may be probably due to the structural changes occurring during development of parasites which could affect the immune response in host, which has also been suggested in case of nematodes [33]. Results show that antigen prepared from immature stages collected from small intestine when employed for ELISA was found to be more immunogenic as evident by the significant increase (*P* < 0.001) in the IgG response and maximum mean difference of IgG in comparison to control group and thus could be a better candidate for early diagnosis of disease. Further, ImRAg and AAg also showed promising results and detected significant increase in IgG levels which could be further exploited for immunodiagnosis, whereas McAg was found to be totally unsuitable for the purpose. Similarly for immunodiagnosis of amphistomosis, adult fluke antigen has been used to detect antibodies at 8 days postinfection (PI) against* P. cervi* [34] and 15 days PI [35] in sheep and goat. Boch et al. [36] detected antibodies against* P. cervi* in calf 8–24 days PI with peak titre of 1 : 512 at 24–50 days PI which persisted up to the stage of egg excretion. Diaz et al. [11] analyzed IgG antibody response against* Calicophoron daubneyi *in cattle utilizing ELISA and found a notable IgG response in naturally infected cattle.

Results of the present study show that developmental stage antigens are very important in the immunodiagnosis of immature amphistomosis in the early stage. Further, identification of immunodominant polypeptides of the most immunogenic (ImIAg), their purification, and large scale validation by use in immunoassays like ELISA need to be carried out with sera collected from field, slaughter house, and experimentally infected animals to work out the sensitivity and specificity. Thus antigens derived from immature stages can lay the possible foundation for development of a serological kit for early diagnosis of amphistomosis in near future.

## Figures and Tables

**Table 1:** Comparison of IgG response against different stage specific antigens of *Paramphistomum  epiclitum *in ELISA by Tukey's test (versus noninfected control).

Antigen	*P* value	Mean difference	95% confidence limit
McAg	>0.05	0.02911	−0.04758 to 0.1058
ImIAg	<0.001	0.1838	0.1071 to 0.2605
ImRAg	<0.001	0.1164	0.03975 to 0.1931
AAg	<0.01	0.1026	0.02586 to 0.1792
